# DailyCog: A Real-World Functional Cognitive Mobile Application for Evaluating Mild Cognitive Impairment (MCI) in Parkinson’s Disease †

**DOI:** 10.3390/s21051788

**Published:** 2021-03-04

**Authors:** Sara Rosenblum, Ariella Richardson, Sonya Meyer, Tal Nevo, Maayan Sinai, Sharon Hassin-Baer

**Affiliations:** 1 The Laboratory of Complex Human Activity and Participation (CHAP), Department of Occupational Therapy, University of Haifa, Haifa 3498838, Israel; 2 Department of Industrial Engineering, Jerusalem College of Technology, Jerusalem 93721, Israel; mayasin295@gmail.com; 3 Department of Occupational Therapy, Ariel University, Ariel 40700, Israel; sonyam@ariel.ac.il; 4 Movement Disorders Institute, Sheba Medical Center, Ramat-Gan 5262000, Israel; Tal.Nevo@sheba.health.gov.il (T.N.); sharon.hassin@sheba.health.gov.il (S.H.-B.); 5 Department of Neurology, Sheba Medical Center, Ramat-Gan 5262000, Israel; 6 Sackler Faculty of Medicine, Tel-Aviv University, Tel-Aviv 6997801, Israel

**Keywords:** eHealth, mobile healthcare, mild cognitive impairment, Parkinson’s disease

## Abstract

Parkinson’s disease (PD) is the second most common progressive neurodegenerative disorder affecting patient functioning and quality of life. Aside from the motor symptoms of PD, cognitive impairment may occur at early stages of PD and has a substantial impact on patient emotional and physical health. Detecting these early signs through actual daily functioning while the patient is still functionally independent is challenging. We developed DailyCog—a smartphone application for the detection of mild cognitive impairment. DailyCog includes an environment that simulates daily tasks, such as making a drink and shopping, as well as a self-report questionnaire related to daily events performed at home requiring executive functions and visual–spatial abilities, and psychomotor speed. We present the detailed design of DailyCog and discuss various considerations that influenced the design. We tested DailyCog on patients with mild cognitive impairment in PD. Our case study demonstrates how the markers we used coincide with the cognitive levels of the users. We present the outcome of our usability study that found that most users were able to use our app with ease, and provide details on how various features were used, along with some of the difficulties that were identified.

## 1. Introduction

Parkinson’s disease (PD) is the second most common progressive neurodegenerative disorder, affecting about 1% of the elderly population, and its multifaceted motor and non-motor afflictions have profound effects on patient functioning and quality of life [1,2]. Among the non-motor symptoms of the disease, both cognitive impairment (CI) and dementia and their implications for patient quality of life have been increasingly recognized [2,3]. Studies show that the onset of PD-specific pathology in the nervous system may precede the first appearance of classical motor features by several years. Mild cognitive impairment (MCI) in PD patients without dementia is present in 25% of newly diagnosed patients [4]. The detection of PD prior to the emergence of motor manifestations is of great importance [1], as MCI in PD patients may have a greater effect on the individual’s daily function abilities and thus their emotio-socio-behavioral status [5]. However, the question of how to detect MCI in a phase in which they are still functionally independent, not dependent on their caregivers, is still unclear [6].

The International Parkinson’s and Movement Disorder Society (MDS) defined diagnostic criteria guidelines for PD-MCI [7]. Nevertheless, there are many questions as how to practically apply the PD-MCI diagnosis [8]. Although formal neuropsychological testing is the mainstay of MCI diagnosis, researchers have criticized using these assessments as measuring specific cognitive domains, not designated specifically for PD, due to lack of standardization and cutoff scores [4,7].

Deficits in executive functions (EF), memory, visual–spatial abilities, and psychomotor speed [3,9] have been mentioned as indicative of a possible cognitive underlying mechanism, as well as relationships with daily function abilities. Furthermore, it was found that activity performance time indeed reflects EF abilities [10,11,12]. As medical diagnoses and clinical judgments are typically derived from changes of condition [13], evidence from daily functioning is usually obtained by standardized self-report scales, to find the best markers of the initial stage of the cognitive decline and for monitoring progression. Thus, an assessment for early diagnosis and detection of PD-MCI should include real-world tasks and self-report questions which may enable the identification of those cognitive deficits through daily functioning in a real-life environment.

Smartphones enable “healthcare in the pocket” and are becoming an increasingly important platform for delivering health interventions [14]. Smartphones offer a valid, feasible and acceptable method for collecting patient health-related data [15,16,17,18]. The number of healthcare mobile applications used by people with varied healthcare needs is vast and constantly expanding [16,19]. Specifically, smartphones have previously been used to collect data for the study of PD. These studies focused mainly on the motor skill [20,21,22,23] or speech [24] manifestations of the disease. Sometimes a combined approach was used for the detection of MCI during progressive stages of PD, and cognitive aspects were detected alongside falls and freezing [25].

Since we focus on the cognitive aspects of PD one might consider apps designed for patients with dementia [26,27]. However, while dementia has clearly presented symptoms that enable straightforward sensing and evaluation, in PD-MCI the clinical presentation is less profound and harder to sense and measure.

Several very impressive projects already described the development of monitoring systems for people with MCI. Among these are INLIFE (Independent Living support Functions for the Elderly) cofounded by the European Union [28], HELMA eHealth monitoring application [29] and the Remote Home Monitoring System to Support Informal Caregivers of People with Dementia [30]. The main global aim of those projects is to support the elderly with MCI in a variety of daily activities through supporting their caregivers and enabling improved service to the elderly. Our purpose is to describe, in detail, a specific monitoring app for detecting real-life activities in people’s homes, through their actual performance when they are still independent, without the involvement of their caregivers. Our aim is to detect the early signs of PD-MCI through the software’s markers, built upon tasks that require EF abilities, visual–spatial abilities, and psychomotor speed, without the need to use other in-home sensors or a caregiver’s involvement. There seems to be agreement, found in several survey studies, that while the need for MCI self-assessment tools is increasing, the existing apps are of uncertain quality and more studies are required [31,32,33].

Our application focuses on the cognitive aspects of PD-MCI, while offering an engaging and realistic environment. We target PD-MCI users (not caregivers), and aim to detect MCI using tasks which reflect cognitive abilities through daily functional performance in the early stages of PD. We based our design on studies that have pointed to the association between MCI to deficits in executive functions (EF), memory, visual–spatial abilities, psychomotor speed, and performance time [3,9,10,11,12]. Furthermore, special attention to the question of technology acceptance, adaptability and accessibility among the older population was addressed in the current study, based on previous literature [34,35,36,37] and on our team’s clinical and experimental experience.

The benefits of implementing a smartphone assessment application are both theoretical and clinical:

1. Valuable information about cognitive abilities through small events and feelings related to daily function, which are often lost, will be captured in real time and in a real functional environment (the home) thus reflecting reality.
2. The collected data may provide better insights into daily events/tasks/measures, which present the best descriptors of cognitive decline, as well as the relationships between them.
3. Analyzing such data may lead to an improved definition of PD-MCI features that could assist clinicians in identifying and detecting PD-MCI for focused intervention methods and further research.
4. There is no need to trouble the patient to go to the clinic for evaluation.
5. The physician can receive information prior to a medical appointment and map out the patient’s cognitive status.

The current study designed and implemented the DailyCog application on a smartphone for capturing and reporting this important information and enabling home healthcare. An earlier version of our work appears in Richardson et al. [18] and describes, in short, the initial guidelines of our design. In this paper, we describe the DailyCog application in detail, in its final form. Furthermore, we performed and describe a user study with two purposes. The first, a case study performed on two patients to demonstrate the feasibility of our application for MCI detection and monitoring. The second, a usability study performed on 36 patients with PD-MCI to better define our app and discover the strengths and weaknesses of our design for the detection of MCI. The results of our case study and then usability test are presented and discussed.

This paper introduces the DailyCog smartphone application. In Section 2 we present our design and describe DailyCog. Our user study is presented in Section 3 along with a detailed description of the participants, a case study and a discussion on usability based on our experiments, conclusions follow in Section 4.

## 2. DailyCog

### 2.1. DailyCog App Description

DailyCog is a smartphone application developed for phones with an android operating system. Impairments in different cognitive domains of MCI may be associated with reduced performance in different aspects of daily functioning. For that purpose, we developed scenarios of real-life situations to reflect day-to-day activity performance characteristics as a source of knowledge about the individual’s cognitive control ability. Addressing the assignments in these scenarios will require the cognitive domains mentioned above (EFs, visual–spatial abilities and memory skills), found to be related to deficient daily function capabilities and include time measurements of activity performance to reflect EF abilities.

The application is composed of a training task and two evaluative tasks. The training task is designed to familiarize the users with the application and the types of tasks they will be required to perform. The two evaluative tasks are to be performed in the home environment at preset intervals (detailed in Section 3.1) and are designed to reflect similar EF abilities, enabling a comparison of functionality over time. The input inserted by the user during their performance is recorded, as are other hidden measures such as the duration of the task performance.

#### 2.1.1. Task Description

The two tasks we designed are:

1. Preparing a *hot drink*.
2. Preparing a *shopping list*.

These tasks are both considered everyday functional tasks, which can be performed at home and require executive functions abilities, visual–spatial abilities, and psychomotor speed [3,13]. They are similar in that they involve planning and collecting several items, which are sorted in a list, following which the task is carried out and photographed by the participant using the app. The similarity of the tasks from the point of view of their functional and cognitive requirements, as well as the time they take, is an important factor, as it enables the comparison of patient’s functional ability over time. We describe the first DailyCog task in detail. The second task is similar in structure but focuses on a different functionality.

**Figure 1:**
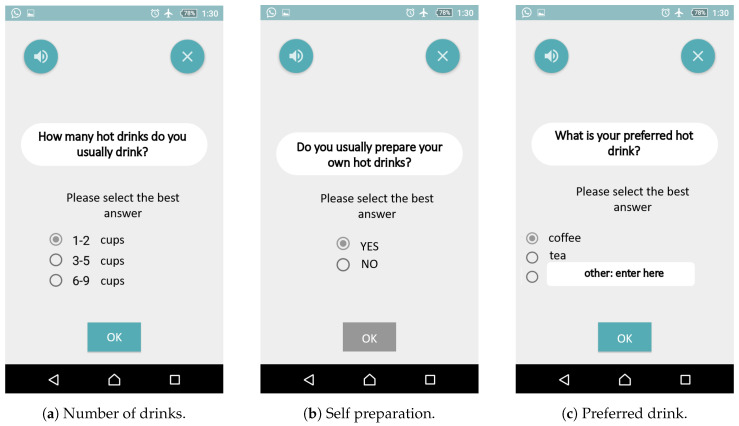
Introductory questions.

In the *hot drink* task, users are guided through the process of preparing a hot drink. They are first given a set of instructions and asked to confirm whether they have free time to perform the task, then they are asked how many hot drinks they usually drink each day, whether they usually prepare their own drink, and what they like to drink. Screenshots of these instructions and questions are shown in Figure 1.

Next they are guided through the process of preparing a hot drink. They are asked how long they expect the preparation to take.

**Figure 2:**
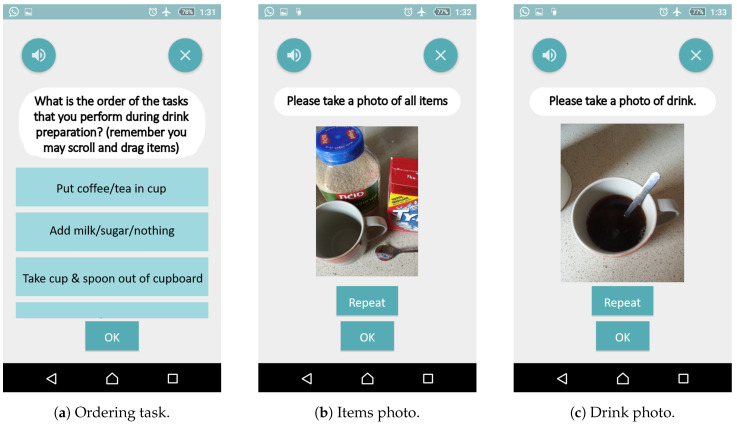
Examples of sub-tasks performed during drink preparation.

The most complex task is to *order* a list of functionalities that are used in drink preparation. DailyCog presents a list of items such as: add milk, boil water, stir, take cup out, put coffee in cup etc. These functions appear in boxes as shown in Figure 2a and need to be sorted in a logical order. The list is longer than most screens and requires the user to be able to scroll down. Ordering is performed using “drag and drop”.

Once the ordering is completed, the user is advised to boil the water and take out all the items needed to prepare the drink, place them on a countertop and to enter the number of items into the DailyCog app. After items are placed on the countertop, DailyCog requests that a photo be taken of all items, as shown in Figure 2b. Please note that a photo can be taken several times. The number of attempts is recorded. When the photo has been taken, the user is asked if the water has boiled, advised to actually prepare the drink and then to take a photo of the drink as shown in Figure 2c, and again the number of attempts made is recorded.

**Figure 3:**
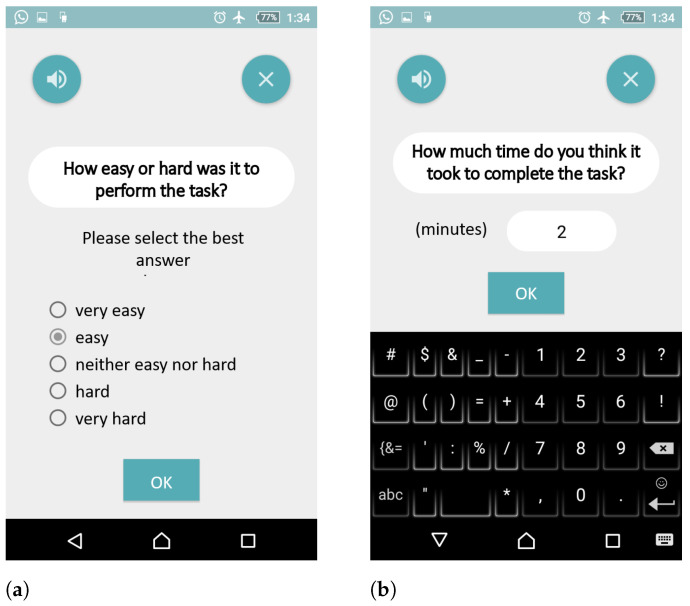
Self-evaluation: as part of the task (**a**,**b**).

Finally, the user is asked how easy the preparation was (Figure 3a), and how long he/she estimates that it took to prepare the drink (Figure 3b). The user is thanked, and then asked to fill out a self-evaluation questionnaire, as described in Section 2.1.2.

The second task is similar in structure to the *hot drink* task, but involves preparing a *shopping list*. In both tasks the user is required to answer questions about time estimates, to put items in a defined place and photograph them, to *order* a list of the functions that need to be performed, and to answer the same self-evaluation questionnaire. This repetitive structure performed at different time intervals is designed to enable tracking of the users’ cognitive condition over time. Please note that we focused on a specific set of limited and well-defined tasks to enable a controlled experiment that is similar to evaluations performed by clinicians. This is not an attempt to capture all the activities performed by users, as is sometimes done in Ambient Assisted-Living settings that monitor all activities.

#### 2.1.2. Self-Evaluation

The final part of DailyCog includes a self-evaluation questionnaire, comprising selected items from the DLQ (Daily Living Questionnaire [38]). The items are not part of the tasks. Rather, they document the user’s self-reported cognitive state, providing another layer in the diagnosis. The self-evaluation questionnaire is given in both stages of the experiment, once after completion of the *hot drink* task, and then again after completion of the *shopping list* task. The items we used relate to how the users rank their ability to think, to function, to do what they need or want to do, and how their tasks and responsibilities have changed since becoming ill. The DLQ scores range from 1 (excellent/no change) to 5 (poor/completely changed).

#### 2.1.3. Data Collection

DailyCog collects two kinds of data. First, all data entered by the user is collected: the times the user enters, the list *ordering*, the photos taken etc. Secondly, there is a set of hidden markers collected during use. For example, the time that the user spent on each screen, the actual time the user spent making the drink, flipping back and forth between screens. There is also an option to ask for help, or to have the text on a screen read aloud. Any use of these activities is logged for future analysis. A complete list of all the data we collected sub-task can be found in Table 1. The sub-tasks for which we recorded the time duration are tagged with a ‘Y’.

### 2.2. DailyCog Design Considerations

In the development of DailyCog, we undertook several design considerations. When targeting patients with PD-MCI one must consider usability and acceptance for this population [34,35,36,37,39]. For example, one should minimize complexity and choice, reduce the number of buttons and options to avoid confusion, make the visual interface easy to understand by using high contrasts, text needs to be large enough to read easily, etc.

One of the features that we included to enable ease of use was the *audio* feature. The user can have the text on a screen read aloud. This feature should help any user who has trouble reading the instructions. Use of this feature is obtained by pressing on the microphone icon that appears at the top of all the screens. Users were advised to use as little help as possible. All usage of this help feature was recorded for analysis.

We also had to decide what measures DailyCog would record. On the one hand, we wanted measures that are easily obtained. On the other hand, we also wanted measures that are easy to analyze. Most importantly, we wanted measures that had a high expectation of being good MCI markers. Sometimes these attributes went hand in hand. For example, consider the *time* measures: We ask the user to evaluate how long the drink preparation will take, how long it took, and we also record the actual time it took from start to end. These measures are both easy to record and to analyze.

Sometimes there is tension between collection and analysis. For example, the *ordering* task from Figure 2a is not trivial to analyze, as there are several possible correct answers. For instance, obviously, one must take the cup out of the cupboard before pouring hot water into the cup. However, one may choose to add the teabag before or after pouring the water. There may be some answers that are preferred to others, such as putting the water in before the milk, but switching the order of these actions is very different to pouring the water before the cup is placed on the counter! Aside from the cognitive aspect of what is considered a correct answer, there is also the technical question of how to compare and grade the different answers. However, even though this task is complex to analyze, it would most likely make a good predictor for MCI. The complexity of the task itself, the cognitive skills required alongside the motor skills used, and the interaction between them, may capture differences between users that we hope will be good markers for MCI. For this reason, we designed and included this complex task.

External disturbances while performing our tasks are problematic, as they may affect the outcome of our experiment. For example, incoming calls on the phone during performance would affect the time taken to perform the tasks, concentration, and perhaps the performance of the user. We addressed this issue by having the user select “airplane mode” before beginning the experimental task to minimize external phone activity. This is shown on all the screenshots where one can see that the phone is in “airplane” mode. We remind the user to exit “airplane” after the task is completed. Although using the “airplane mode” improves the chances that our users can concentrate on performing the task and disturbances such as incoming calls or messages are avoided, we cannot completely eliminate distractions and discuss this further in Section 3.3.

## 3. User Study

We carried out a user study on patients with PD who performed the designed tasks in their homes. The aim of this study was both to demonstrate and evaluate the feasibility of using DailyCog for PD-MCI, and to raise issues that require future fine tuning before performing large scale experimentation. We begin by discussing the inclusion criteria for our study. Then we proceed to describe a case study for two patients with various degrees of MCI. We also discuss issues of usability learned from all participating users.

### 3.1. Study Design and Selection Criteria

The DailyCog application study was conducted in 3 phases. The first phase is a training phase. The participants are advised on how to use DailyCog and perform a training task under the guidance of a trained practitioner in the office. This task is used only for training and is not analyzed as part of the evaluation. The next two tasks are the evaluation tasks described in Section 2.1. The two evaluation tasks were carried out with an interval of 3–7 months between each task. The two tasks were performed using a smartphone in the user’s home, without the supervision of a practitioner. The activities performed by the users during task performance are collected automatically from the smartphone and uploaded to a protected server for analysis. Details on the tasks in the DailyCog application are described in Section 2.1, with special design considerations described in Section 2.2.

The study included 36 participants with PD aged 40 to 80 years, recruited at the Movement Disorders Institute in the Chaim Sheba Medical Center, Ramat Gan, Israel. They were functionally independent and lived in their private homes or assisted-living facilities. See Rosenblum et al. [6] for more details.

### 3.2. Case Study

We present two case studies chosen from among the participants representing different cognitive abilities. We used the MoCA test scores [40] of the users (taken as part of the pretrial evaluation) to differentiate between high/low levels of MCI. Lower MoCA scores point towards a higher degree of cognitive impairment. It must be noted that while there is an ongoing discussion on the use of the MoCA test for evaluating PD-MCI [6], this is, to date, the standard measure used. User-A scored MoCA = 21 and User-B scored MoCA = 27. Both users completed the tasks. They performed the *ordering* tasks correctly, uploaded all the necessary photographs etc. We also include results for the full 36 users. These results are presented as the average and standard deviation over all users. We did not obtain statistical significance for the differences between users with high and low MoCA scores (we used 24 as the cutoff), all *p* values were larger than 0.05. The *p* values for Total time were 0.85 (hot drink) and 0.11 (shopping list). For Order time they were 0.85 (hot drink) and 0.53 (shopping list), and for the self-evaluation scores they were 0.37 (hot drink) and 0.96 (shopping list). These results support the findings in [6].

Table 2 compares the times that it took the users to perform tasks as related to their MoCA scores. We measure two time intervals. *Total time* is the time it took the user to complete the whole task. *Order time* is the time it took to perform the *ordering* task (which is part of the task, as described in Section 2.1). The times are compared across the two tasks, the *hot drink* and the *shopping list* tasks. The tasks are performed with over six months between them. As shown in Table 2, User-A is slower than User-B on both tasks (*shopping list* and *hot drink*) and for both time measurements (*Total time* and *Order time*). This finding is consistent with the MoCA scores of both users, since User-A, who suffers from a more substantial degree of MCI than User-B, needs more time to perform task activities. Furthermore, there seems to be some deterioration in the abilities of User-A as time goes by. When we compare the two tasks that were performed over approximately a 6-month period, we see that User-A requires more time to complete the second task (*shopping list*), than it took him to complete the first task (*hot drink*). In contrast, User-B requires almost the same amount of time to complete the two tasks. Please note that both the *Total time* and the *Order time* increase by a factor of approximately 1.5. The fact that when *Order time* increases the *Total time* increases as well is not surprising, as the total time includes the Order time. It is, however, interesting that the factor between the times is consistent, perhaps pointing towards an overall slowness in performance in all activities, and not only the *ordering* activity which requires a high degree of coordination and cognition compared to some of the other parts of the task.

Table 3 presents the results for the self-evaluation questionnaire, as described in Section 2.1.2. The questions reflect the users’ feeling on how they view their cognitive state and their functional abilities both inside and outside home. There are 6 questions that the users answer. The questions are answered immediately after completing the tasks. Answers are rated from 1–5, where 1 is Excellent and 5 is Poor. Thus, a higher grade reflects a higher degree of MCI.

As with the results in Table 2, the results in Table 3 reflect a deterioration for User-A between the performance of the two tasks. For User-B the scores are almost completely stable. The same ratio of 1.5 that was seen for the task performance times is again apparent for the average score of User-A. This may imply that the self-evaluation is correlative to the time measure. This finding contributes the indication that time measurements for our tasks reflect the self-reporting. It is, however, interesting to note that while the trend of the scores correlates to the trend in times, looking at the absolute value of the scores is surprising. User-B, who scores higher on the MoCA and spends less time performing both tasks than User-A has a self-report that presents a higher degree of impairment. Perhaps this is because the self-report is very subjective, and, as opposed to time measurements or the MoCA evaluation that are objective, a self-report also takes emotional state and personal feelings into account. This would suggest being careful with the self-evaluation, and using the score more as a comparative test where trend rather than the raw score is the important measure.

### 3.3. Usability Study

Data collection was completed by 36 users. We set out to determine whether DailyCog can detect deterioration of MCI. Since the abilities of the users were taken into consideration during the app design, most of our participants reported that they found our app easy to use and instructions easy to follow, and they were happy to complete the experiment. Photographs taken as part of the task provided proof that the users followed the tasks to completion and performed the requested steps. Our case study in Section 3.2 demonstrates encouraging results. Although a broader study is necessary in order to collect enough information for a more comprehensive analysis, perhaps enabling the use of machine learning methods, the preliminary study provides evidence of our ability to capture various aspects of PD-MCI.

**Figure 4:**
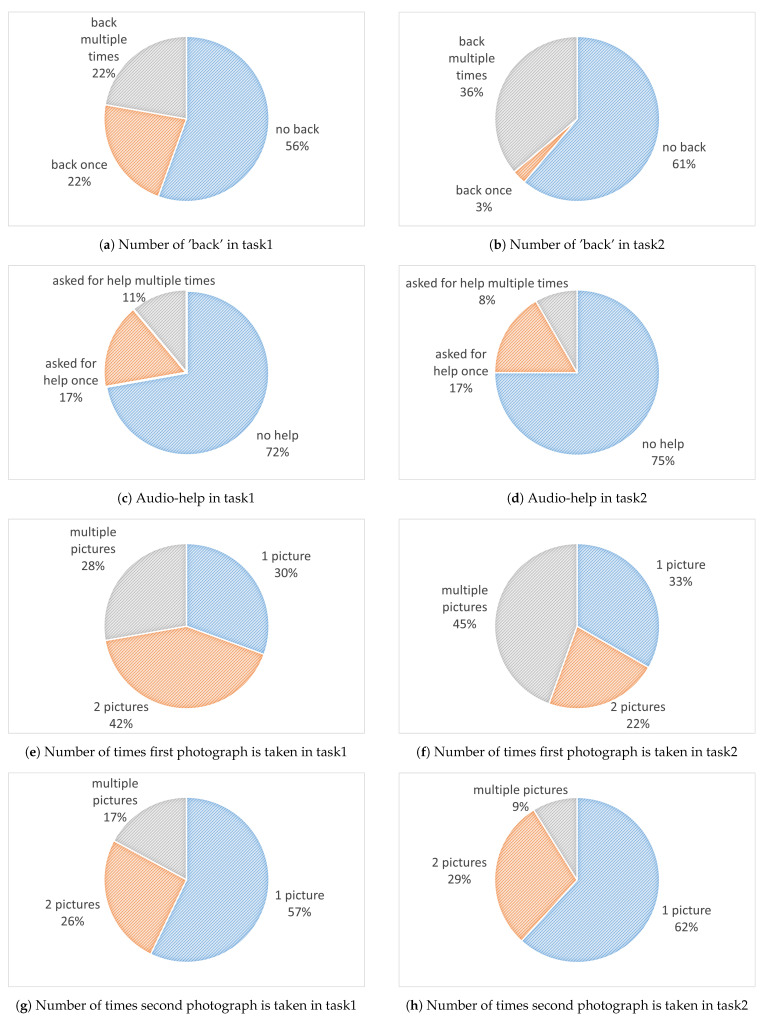
Usability study outcomes.

Some of the results obtained in this study are presented in Figure 4. The left column describes outcomes from the first task and the right column from the second task. Figure 4a,b show that more than half the users completed the tasks without pressing the ‘back’ button. However, around 40% of the users made use of this button during the first task, and their number increased in the second task. The help button that provides audio explanation was not used by most users. This indicates that most users understood the tasks they were asked to perform. However, some of the users needed the extra assistance. Figure 4c,d show that 17% of the users made use of the audio-help during both tasks.

In each of the two tasks, there are two photographs taken. The number of attempts made are shown in Figure 4e–h. Perhaps the most interesting finding regarding the photos is that in both tasks the second one was easier to take. In the first attempt approximately 30% of the users made one attempt, the others needed more. However, for the second photo close to 60% managed to take the photo with a single attempt.

We encountered various obstacles in our study, as did many other researchers in these domains [35,36,37]. Although these issues may seem obvious in hindsight, it is often the case that they are not, and caregivers are often needed to manage the application use, as in Cossu-Ergecer et al. [29]. We discuss the main difficulties that we encountered to contribute to the planning of similar future studies.

The first difficulty we encountered was hesitance and insecurity in using an app by some of our participants. We tried to minimize this by selecting participants who already owned and used a smartphone, and we coached them through a training session that demonstrated all the actions we expected them to perform in the study. Despite these preparations we found that many of our users had trouble during the experiment and required support from our staff. Often this support was needed to give the user confidence, rather than to solve a usage problem. We did not encounter problems with the smartphone touch, as DailyCog is aimed at users that at the very initial stages of PD, before significant manual dysfunction manifest. This causes the possibility that DailyCog may not be suited for users with progressive PD.

The support given by our staff to a small number of participants raises the question of how to evaluate the experimental data in experiments such as ours. Once there is intervention by someone other than the participant, measures such as time and performance might be contaminated. Users sometimes chose to report this to us, and we could exclude them from our study. We are confident that most of the other users performed the experiment alone as required. However, we draw attention to this point as to contribute to the planning of future studies.

Since the experiment ran over a course of many months, some users swapped phones during the experiment, resulting in a variety of problems ranging from reinstalling the app (easy to solve) to changing from an android system (that the app was developed for) to an iPhone (resulting in dropping out of the trial).

Since we study functionality in the home environment, we did not have full control over the experiment. We made an effort to control some of the settings, for example by moving the phone into “airplane mode” so that no phone calls or messages would intervene. However, we had no control over factors such as someone ringing a doorbell, or calling a landline, etc. Such events would obviously affect the time measure of our experiment and may not have been reported. Although we want our experiment to take place at home to mimic everyday functionality, we pay the price of losing control over many of the factors. Thus, there is a trade-off between complete control and studying functionality in a natural environment.

We also noticed a possible interference effect. As was to be expected, not all the users displayed deterioration between the performances of the two tasks. This could be explained by the relative stability of MCI levels observed in some patients. However, we noticed that some of the users actually improved; they became better at the tasks and performed the second task faster than the first. Although we were careful to design two different tasks to limit the ‘learning factor’ it would seem that some users did learn. The way to counter the learning factor would be to design tasks with a more significant difference. However, that would come at the price of losing the possibility to compare cognitive states across the tasks. Alternatively, we could randomize the order of task performance among users. We plan to tackle this issue in future work.

## 4. Conclusions

We designed and developed DailyCog to explore a mobile app for detecting and evaluating PD-MCI. This is an important step towards early detection of PD, since MCI is often an early symptom of PD and may have a substantial impact on quality of life.

DailyCog was developed based on clinical experience and familiarity with conventional MCI detection methods. We were led by the concept that markers are hidden in performing everyday tasks. Therefore, performing tasks at home, noting the time taken to perform tasks, the way the task is performed, and what tasks are completed etc., may provide insight into the users’ abilities in real life. The DailyCog app is unique in that it enables participants to perform daily functions in their familiar home environment in a measurable fashion.

DailyCog includes a training task and two everyday tasks: *hot drink* preparation and making a *shopping list*. These tasks are to be performed over predefined time intervals to capture the state of the MCI over time. Aside from the guidance and recording of everyday tasks, DailyCog includes a short self-report questionnaire to enable self-reporting of cognitive decline. The similar structure of the two tasks enables a comparison of the participants’ performances during each task to evaluate their cognitive states.

We conducted a study on users with PD-MCI and presented case study results alongside a usability study. Our case study demonstrated how DailyCog captures both stable and deteriorating patients. We presented results and insights from our usability study. We described the strengths of DailyCog and shared difficulties that should be addressed in future studies with DailyCog or other similar environments. DailyCog was developed with PD-MCI as the main target but is easily usable or adaptable for other similar conditions involving cognitive decline.

## Figures and Tables

**Table 1:** Recorded data and markers.

Marker Name	Description (Task Data Recorded)	Task Time Recorded
date	Date task is performed	-
back press	Number of times the user pressed the “back” button	-
exit press	Number of times the user pressed the “exit” button	-
sound press	Number of times the user asked for audio-help	-
time total	The total time it took to complete the task	-
pic products	The number of photographs of the items that were taken	-
pic beverage	The number of photographs of the prepared drink that were taken	-
free time	Answer—do you have free time?	Y
cups frequency	Answer—How many drinks do you drink	Y
usually make	Answer—Do you usually prepare your own drinks?	Y
preferred beverage	Answer—What do you like to drink?	Y
est prep time	Answer—How long does it take to prepare your drink?	Y
order	The outcome of the *ordering* task.	Y
boil water	Instruction to boil the water	Y
place products	Instruction to place products on counter	Y
pic products	Instruction to take a photograph of products on counter	Y
water boiled	Answer—Has water boiled?	Y
all products	Answer—Are all products ready?	Y
pic beverage	Instruction to take a photograph of prepared drink	Y
difficulty	Answer—How difficult was task?	Y
est performance	Answer—How long do you estimate the task took?	Y

**Table 2:** Comparison of times (seconds) for case study users and All users.

User	MoCA Score	Task	*Total Time*	*Order Time*
A	21	*hot drink*	344.8	65.5
		*shopping list*	527.3	109.6
B	27	*hot drink*	213.5	40.2
		*shopping list*	217.6	43.2
All	23.8 ± 3.1	*hot drink*	493.8 ± 228.3	90.4 ± 66.5
		*shopping list*	438.7 ± 207.1	49.6 ± 41.6

**Table 3:** Comparison of self-questionnaire for case study users and All users.

User	MoCA Score	Task	Average
A	21	*hot drink*	1.7
		*shopping list*	2.5
B	27	*hot drink*	2.8
		*shopping list*	3
All	23.8 ± 3.1	*hot drink*	1.9 ± 1
		*shopping list*	1.7 ± 1.1

## Data Availability

The data presented in this study are available on request from the corresponding author. The data are not publicly available due to the privacy of the participants.

## References

[1] Chahine L.M., Stern M.B., Chen-Plotkin A. (2014). Blood-based biomarkers for Parkinson’s disease. Park. Relat. Disord..

[2] Jankovic J. (2008). Parkinson’s disease: Clinical features and diagnosis. J. Neurol. Neurosurg. Psychiatry.

[3] Aarsland D., Bronnick K., Williams-Gray C., Weintraub D., Marder K., Kulisevsky J., Burn D., Barone P., Pagonabarraga J., Allcock L. (2010). Mild cognitive impairment in Parkinson disease: A multicenter pooled analysis. Neurology.

[4] Barone P., Aarsland D., Burn D., Emre M., Kulisevsky J., Weintraub D. (2011). Cognitive impairment in nondemented Parkinson’s disease. Mov. Disord..

[5] Dubois B., Burn D., Goetz C., Aarsland D., Brown R.G., Broe G.A., Dickson D., Duyckaerts C., Cummings J., Gauthier S. (2007). Diagnostic procedures for Parkinson’s disease dementia: Recommendations from the movement disorder society task force. Mov. Disord..

[6] Rosenblum S., Meyer S., Gemerman N., Mentzer L., Richardson A., Israeli-Korn S., Livneh V., Karmon T.F., Nevo T., Yahalom G. (2020). The Montreal Cognitive Assessment: Is It Suitable for Identifying Mild Cognitive Impairment in Parkinson’s Disease?. Mov. Disord. Clin. Pract..

[7] Litvan I., Goldman J.G., Tröster A.I., Schmand B.A., Weintraub D., Petersen R.C., Mollenhauer B., Adler C.H., Marder K., Williams-Gray C.H. (2012). Diagnostic criteria for mild cognitive impairment in Parkinson’s disease: Movement Disorder Society Task Force guidelines. Mov. Disord..

[8] Rosenblum S., Werner P. (2006). Assessing the handwriting process in healthy elderly persons using a computerized system. Aging Clin. Exp. Res..

[9] Muslimović D., Post B., Speelman J.D., Schmand B. (2005). Cognitive profile of patients with newly diagnosed Parkinson disease. Neurology.

[10] Rosenblum S. (2013). Handwriting measures as reflectors of executive functions among adults with Developmental Coordination Disorders (DCD). Front. Psychol..

[11] Rosenblum S., Aloni T., Josman N. (2010). Relationships between handwriting performance and organizational abilities among children with and without dysgraphia: A preliminary study. Res. Dev. Disabil..

[12] Rosenblum S., Regev N. (2013). Timing abilities among children with developmental coordination disorders (DCD) in comparison to children with typical development. Res. Dev. Disabil..

[13] Jørgensen J.T. (2011). A challenging drug development process in the era of personalized medicine. Drug Discov. Today.

[14] Klasnja P., Pratt W. (2012). Healthcare in the pocket: Mapping the space of mobile-phone health interventions. J. Biomed. Inform..

[15] Boulos M.N.K., Wheeler S., Tavares C., Jones R. (2011). How smartphones are changing the face of mobile and participatory healthcare: An overview, with example from eCAALYX. Biomed. Eng. Online.

[16] Boulos M.N.K., Brewer A.C., Karimkhani C., Buller D.B., Dellavalle R.P. (2014). Mobile medical and health apps: State of the art, concerns, regulatory control and certification. Online J. Public Health Inform..

[17] Ozdalga E., Ozdalga A., Ahuja N. (2012). The smartphone in medicine: A review of current and potential use among physicians and students. J. Med Internet Res..

[18] Richardson A., Rosenblum S., Hassin-Baer S. Multidisciplinary Teamwork in the Design of DailyCog for Evaluating Mild Cognitive Impairment (MCI) in Parkinson’s Disease. Proceedings of the 2019 International Conference on Virtual Rehabilitation (ICVR).

[19] Terry K. (2015). Number of health apps soars but use does not always follow. Medscape Medical News.

[20] Landers M.R., Ellis T.D. (2020). A Mobile App Specifically Designed to Facilitate Exercise in Parkinson Disease: Single-Cohort Pilot Study on Feasibility, Safety, and Signal of Efficacy. JMIR mHealth Uhealth.

[21] Mazilu S., Hardegger M., Zhu Z., Roggen D., Troster G., Plotnik M., Hausdorff J.M. Online detection of freezing of gait with smartphones and machine learning techniques. Proceedings of the 2012 6th International Conference on Pervasive Computing Technologies for Healthcare (PervasiveHealth).

[22] Lan K.C., Shih W.Y. (2014). Early Diagnosis of Parkinson’s Disease Using a Smartphone. Procedia Comput. Sci..

[23] Bot B.M., Suver C., Neto E.C., Kellen M., Klein A., Bare C., Doerr M., Pratap A., Wilbanks J., Dorsey E.R. (2016). The mPower study, Parkinson disease mobile data collected using ResearchKit. Sci. Data.

[24] Zhang L., Qu Y., Jin B., Jing L., Gao Z., Liang Z. (2020). An Intelligent Mobile-Enabled System for Diagnosing Parkinson Disease: Development and Validation of a Speech Impairment Detection System. JMIR Med. Inform..

[25] Lo C., Arora S., Baig F., Lawton M.A., El Mouden C., Barber T.R., Ruffmann C., Klein J.C., Brown P., Ben-Shlomo Y. (2019). Predicting motor, cognitive & functional impairment in Parkinson’s. Ann. Clin. Transl. Neurol..

[26] Sposaro F., Danielson J., Tyson G. iWander: An Android application for dementia patients. Proceedings of the 2010 Annual International Conference of the IEEE Engineering in Medicine and Biology.

[27] Fernández Montenegro J.M., Villarini B., Angelopoulou A., Kapetanios E., Garcia-Rodriguez J., Argyriou V. (2020). A Survey of Alzheimer’s Disease Early Diagnosis Methods for Cognitive Assessment. Sensors.

[28] Kaimakamis E., Karavidopoulou V., Kilintzis V., Stefanopoulos L., Papageorgiou V. (2017). Development/Testing of a Monitoring System Assisting MCI Patients: The European Project INLIFE. Stud. Health Technol. Inform..

[29] Cossu-Ergecer F., Dekker M., van Beijnum B.F., Tabak M. Usability of a New eHealth Monitoring Technology That Reflects Health Care Needs for Older Adults with Cognitive Impairments and Their Informal and Formal Caregivers. Proceedings of the 11th International Joint Conference on Biomedical Engineering Systems and Technologies—Volume 5 Healthinf: Healthinf, INSTICC.

[30] Lentelink S., Tabak M., van Schooten B., Hofs D., op den Akker H., Hermens H. A Remote Home Monitoring System to Support Informal Caregivers of People with Dementia. Proceedings of the 11th International Joint Conference on Biomedical Engineering Systems and Technologies—Volume 5 Healthinf: Healthinf.

[31] Thabtah F., Peebles D., Retzler J., Hathurusingha C. (2020). Dementia Medical Screening using Mobile Applications: A Systematic Review with A New Mapping Model. J. Biomed. Inform..

[32] Charalambous A.P., Pye A., Yeung W.K., Leroi I., Neil M., Thodi C., Dawes P. (2020). Tools for app-and web-based self-testing of cognitive impairment: Systematic search and evaluation. J. Med. Internet Res..

[33] Klimova B. (2017). Mobile phone apps in the management and assessment of mild cognitive impairment and/or mild-to-moderate dementia: An opinion article on recent findings. Front. Hum. Neurosci..

[34] Holden R.J., Karsh B.T. (2010). The technology acceptance model: Its past and its future in health care. J. Biomed. Inform..

[35] Isaković M., Sedlar U., Volk M., Bešter J. (2016). Usability pitfalls of diabetes mHealth apps for the elderly. J. Diabetes Res..

[36] Pang G.K.H., Kwong E. Considerations and design on apps for elderly with mild-to-moderate dementia. Proceedings of the 2015 International Conference on Information Networking (ICOIN).

[37] Li C., Neugroschl J., Zhu C.W., Aloysi A., Schimming C.A., Cai D., Grossman H., Martin J., Sewell M., Loizos M. (2020). Design Considerations for Mobile Health Applications Targeting Older Adults. J. Alzheimer’s Dis..

[38] Rosenblum S., Josman N., Toglia J. (2017). Development of the Daily Living Questionnaire (DLQ): A Factor Analysis Study. Open J. Occup. Ther..

[39] Mayer J.M., Zach J. Lessons learned from participatory design with and for people with dementia. Proceedings of the 15th International Conference on Human-Computer Interaction with Mobile Devices and Services.

[40] Nasreddine Z.S., Phillips N.A., Bédirian V., Charbonneau S., Whitehead V., Collin I., Cummings J.L., Chertkow H. (2005). The Montreal Cognitive Assessment, MoCA: A brief screening tool for mild cognitive impairment. J. Am. Geriatr. Soc..

